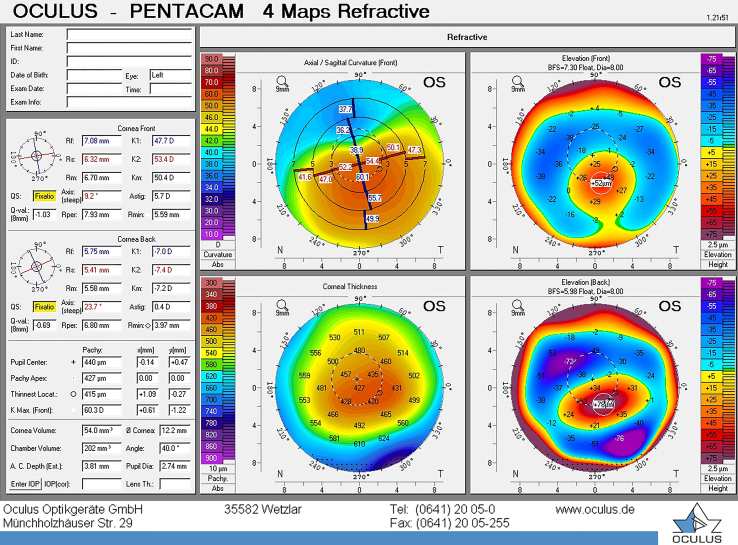# Late-onset corneal edema after customized crosslinking for progressive keratoconus

**DOI:** 10.1016/j.ajoc.2024.102090

**Published:** 2024-06-16

**Authors:** Magali M.S. Vandevenne, Tos T.J.M. Berendschot, Nienke Visser, Mor M. Dickman, Rudy M.M.A. Nuijts

**Affiliations:** Maastricht University Medical Centre+ University Clinic of Ophthalmology, the Netherlands, P.O. box 5800, 6202 AZ Maastricht, the Netherlands

**Keywords:** Keratoconus, Customized, Corneal crosslinking, Edema, Endothelial cell loss

## Abstract

**Purpose:**

We describe a patient after customized crosslinking (CXL) for progressive keratoconus who developed corneal edema with spontaneous resolution.

**Observations:**

A 24-year-old male with progressive keratoconus of the left eye underwent a customized CXL procedure with a total energy of 10 J/cm^2^ for 16.4 minutes. Preoperative corrected distance visual acuity (CDVA) was 20/30 with a maximum keratometry (K)-value of 58.6 diopter (D) and the thinnest point measured 414 μm. The preoperative endothelial cell density (ECD) was 2414 cells/mm^2^. During treatment, corneal thickness was 325 μm after epithelial debridement and 375 μm after the application of 0.1 % riboflavin containing HPMC. After the treatment, antibiotic and steroid drops were prescribed for 5 days and 3 weeks, respectively. At the 1-month post-CXL visit the patient had no complaints, visual acuity and clinical examination showed no irregularities. At the 4-months post-CXL visit the patient complained of blurry vision. The CDVA was 20/100 and slit-lamp examination showed microcystic corneal edema. The corneal thickness at the thinnest point measured 440 μm. One month later the edema had resolved spontaneously and CDVA had restored to 20/25. Corneal thickness at the thinnest point measured 415 μm, the ECD was 1514 cells/mm^2^ and confocal microscopy showed normal structural changes in the anterior stroma after CXL, with the demarcation line located at a depth of 414 μm, just above the corneal endothelium.

**Conclusions and importance:**

We report a case of corneal edema following customized CXL with endothelial cell loss that resolved spontaneously. We recommend either adhering to a minimal stromal thickness of 400 μm before administering UV-A irradiation, using a contact lens or adjusting the irradiation to prevent this complication.

## Highlights

• Corneal edema after customized crosslinking.
• Spontaneous resorption of corneal edema after customized crosslinking.
• Endothelial cell loss with corneal edema after customized crosslinking.
• ‘The 400 μm rule’ for minimum corneal thickness.

## 1 Introduction

Keratoconus, an ectatic corneal disease typically presenting bilaterally, is characterized by its progressive nature, involving corneal thinning, irregular astigmatism, and a decrease in visual acuity.^1^ The estimated prevalence of keratoconus in the Netherlands is 1 in 375.^2^ Normally, the disease begins during puberty and progresses into the mid-40s.^2^ When keratoconus is stable, treatment is focused on improving vision with glasses and contact lenses. However, when keratoconus progresses, treatment is indicated.

In 2003, Wollensak et al. introduced corneal crosslinking (CXL) as a minimally invasive treatment to stabilize keratoconus.^3^ This procedure uses riboflavin (vitamin B2) and ultraviolet radiation (UV-A) to halt disease progression. The classic Dresden protocol is an epi-off procedure where the cornea is impregnated with riboflavin 0.1 % solution (20 % dextran) for 30 minutes. Subsequently, the cornea is irradiated with 3 mW/cm^2^ UV-A light for 30 minutes, giving a total energy of 5.4 J/cm^2^. In recent years, alternative protocols have been introduced, including accelerated and epi-on crosslinking.^4^^,^^5^ More recently, studies have evaluated customized CXL or topography-guided CXL, which has been shown to be safe and effective in halting the progression.^6^, ^7^, ^8^, ^9^, ^10^ It is hypothesized that selectively targeting the most affected part of the cornea may result in a similar outcome (i.e. stabilization of corneal curvature) as targeting the entire cornea.^11^ Furthermore, three potential benefits have been described: (1) a faster recovery (e.g. increased corneal reepithelialisation), (2) stronger flattening of maximum keratometry and (3) better visual outcome.^6^^,^^8^, ^9^, ^10^

In general, CXL has been shown to be a safe procedure. Nevertheless, adverse events after CXL have been described, such as corneal haze, microbial keratitis, and delayed epithelial healing.^12^ A relatively rare adverse event is corneal edema. It usually occurs within a couple of days after the procedure and is temporary, although cases with persistent edema have been described.^13^^,^^14^ A possible cause of edema after CXL is endothelial cell damage.^15^

This report describes a case of corneal edema after customized CXL in a patient with progressive keratoconus.

## 2 Case report

A 24-year-old male was referred to our clinic with forme fruste keratoconus in his right eye and severe keratoconus in his left eye. In the past, the left eye was treated for amblyopia. Medical history was positive for eye rubbing. During the examination, the corrected distance visual acuity (CDVA) was 20/25 in his right eye and 20/30 in his left eye. Slit-lamp examination revealed a clear cornea in both eyes with a few Vogt's striae in the left eye. The intraocular eye pressure was 11 mmHg in both eyes. Scheimpflug tomography measurement (Pentacam HR, Oculus, Wetzlar, Germany) showed a maximum keratometry value (Kmax) of 57.5 diopter (D) and a corneal thickness at the thinnest point of 422 μm in the left eye (Fig. 1).

**Fig. 1:**
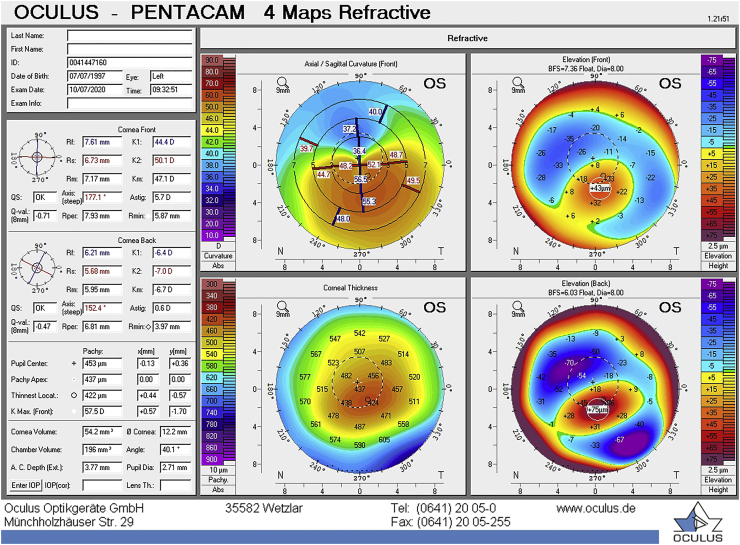
Refractive display by the Pentacam HR showing keratoconus in the left eye at the first outpatient visit.

**Fig. 2:**
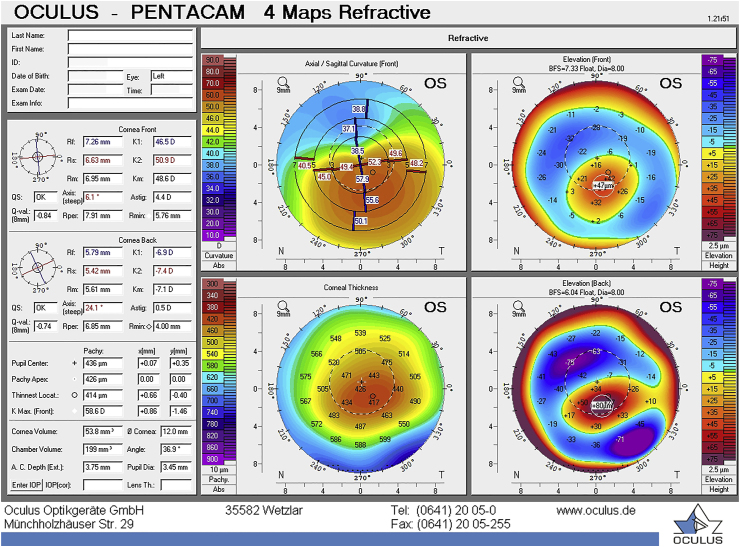
Refractive display by the Pentacam HR showing progressive keratoconus after 12 months in the left eye, Kmax increased with 1.1 D and thinnest pachymetry decreased with 8 μm.

The patient came for a control visit every 6 months. At his follow-up appointment, 12 months after his first visit, progression in his left eye was observed. Progression is defined as an increase in Kmax of >1 D, a 10 % decrease in corneal thickness, or a 1 D change in refractive spherical equivalent.^16^ The Kmax was 58.6 D (in line with the criteria for progression) and the corneal thickness at the apex was 426 μm and at the thinnest point was 414 μm (Fig. 2). The endothelial cell density (ECD) in his left eye was 2414 cells/mm^2^ (TOPCON SP-3000P, Topcon Corporation, Tokyo, Japan).

The patient was scheduled for Scheimpflug tomography-guided customized CXL. The treatment pattern was centered on the cone, where the cone location is defined as a combination of the thinnest corneal point, and the maximum anterior and posterior elevation. The average of these 3 points is calculated and functions as the center of the treatment pattern. The treatment pattern itself has 3 concentric circles. The diameter of the innermost circle is 4 mm, of the middle circle 5.2 mm, and of the outermost circle is 6 mm. The innermost circle receives the highest amount of energy, 10 J/cm^2^, which is equal to a fluence of 10 mW/cm^2^ for 16 minutes and 40 seconds. The middle circle receives 7.2 J/cm^2^ and the outermost circle receives 5.4 J/cm^2^. The procedure is done with the Avedro Mosaic CXL device (Avedro, Inc. Waltham, Massachusetts, United States).

Before the start of the treatment, anesthetic drops (oxybuprocaine 4 mg/ml and tetracaine 5 mg/ml) were given. The epithelium was debrided with alcohol 20 % within the marked treatment zone of 6 mm and the corneal thickness was measured. The corneal thickness after debridement was 325 μm. Subsequently, the cornea was soaked with 0.1 % riboflavin containing HPMC (Vibex Rapid™, Avedro Inc.) reaching a maximal central thickness of 375 μm. Customized irradiation with a total energy of 10 J/cm^2^ was applied and postoperatively, a topical antibiotic (ofloxacin, Trafloxal, 3 mg/g, Bausch and Lomb, Dublin, Ireland) was administered and a bandage contact lens was placed on the cornea.

At the 5-day postoperative visit, the patient had no complaints. Slit-lamp examination showed that the epithelial abrasion was closed, with few epithelial punctate erosions inferior on the cornea. The topical antibiotic drops were stopped and topical steroids (fluorometholon, FML Liquifilm, 1 mg/ml, Allergan, Westport, Ireland) were prescribed for 3 weeks.

**Fig. 3:**
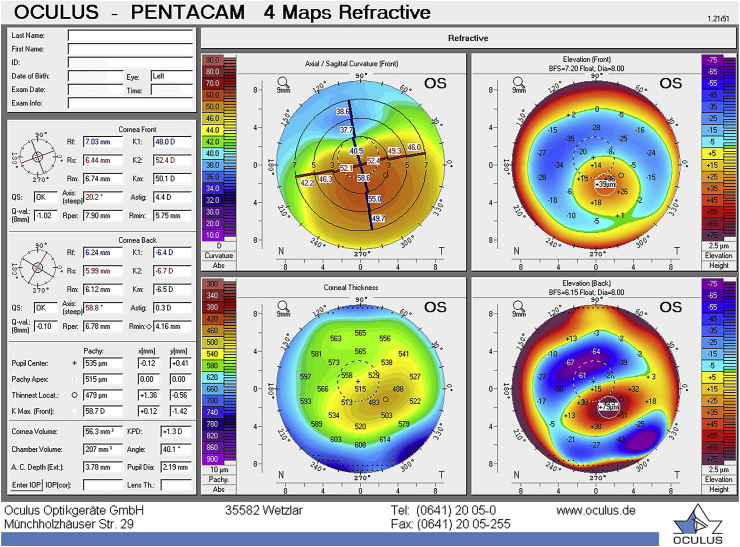
Refractive display by the Pentacam HR showing the left eye 1 month post-CXL.

**Fig. 4:**
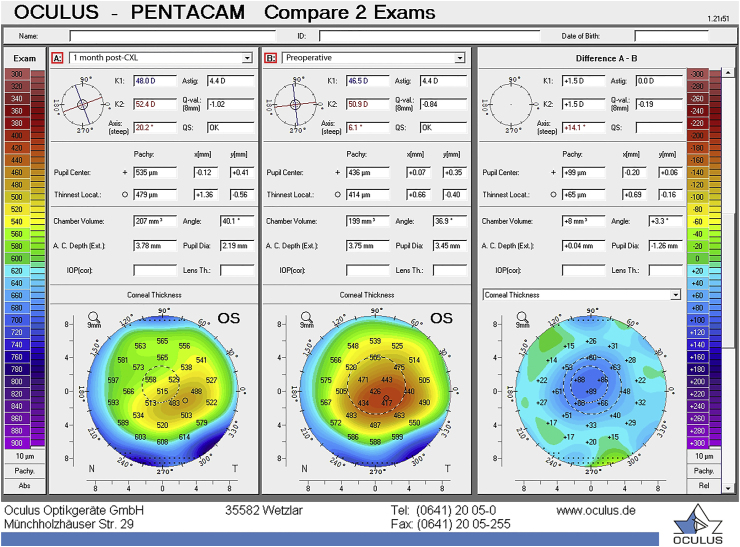
Difference map of 1 month post-CXL (A) and preoperative (B) Pentacam measurement, there is an increase in flat and steep K-values of 1.5 D and a slight increase of maximum K-value of 0.1 D. The corneal thickness at the apex shows an increase of 89 μm and at the thinnest point 65 μm.

**Fig. 5:**
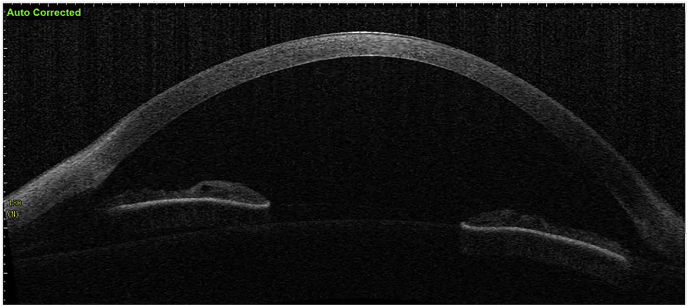
Anterior segment OCT of the left eye 1 month post-CXL, the demarcation line is not visible.

At the 1-month postoperative visit, the CDVA of the left eye was 20/30. The cornea was clear with minor punctate keratopathy inferiorly. There was an increase in flat and steep K-values of 1.5 D (Fig. 3) and a slight increase of maximum K-value of 0.1 D. An increase in K-values one month post-CXL has been shown previously.^17^ The increase in corneal thickness at the apex was 89 μm and at the thinnest point 65 μm (Fig. 3, Fig. 4). Anterior segment optical coherence tomography (OCT) (Tomey SS-1000 CASIA OCT device (Tomey, Nagoya, Japan)) was performed, but a demarcation line was not seen (Fig. 5). The demarcation line is the transition zone between the crosslinked tissue and the non-crosslinked tissue.^18^ According to Mazzotta et al. it correlates with the biomechanical efficacy of the CXL treatment.^19^

**Fig. 6:**
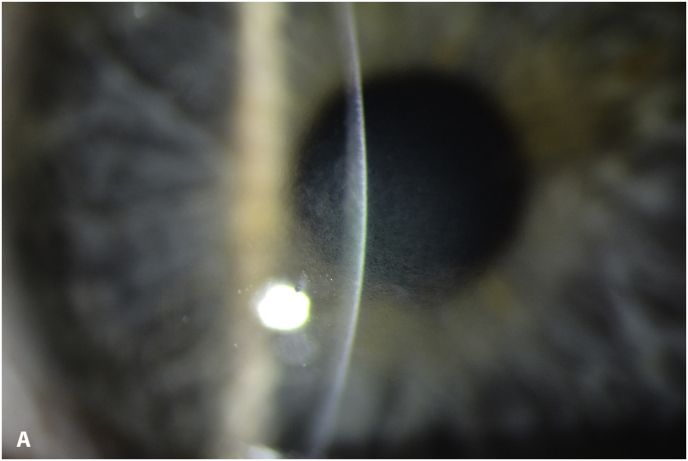

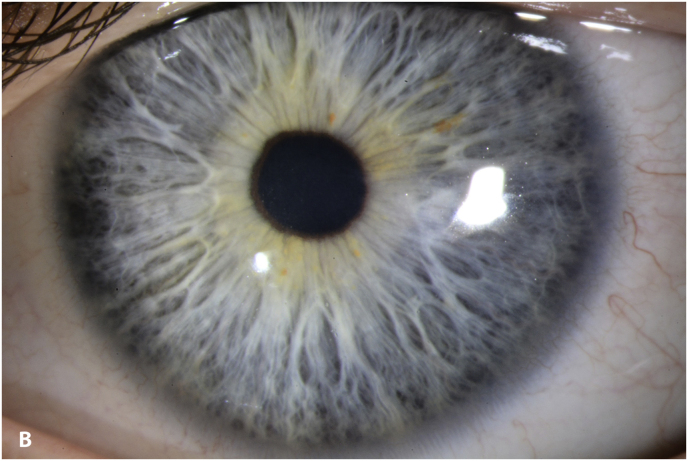
A. slit-lamp image of the left eye 4 months post-CXL showing microcystic corneal edema; B. slit-lamp image of the left eye 5 months post-CXL showing a clear cornea.

At the 4-month follow-up visit the patient complained of blurry vision. An UDVA of 20/200 and a CDVA of 20/100 was measured. Slit-lamp examination showed microcystic edema (Fig. 6a). Corneal thickness was 617 μm at the apex and 440 μm at the thinnest point (Supplementary Fig. 1). Specular microscopy was not possible due to corneal edema. A wait-and-see approach was chosen, no medication was prescribed.

**Fig. 7:**
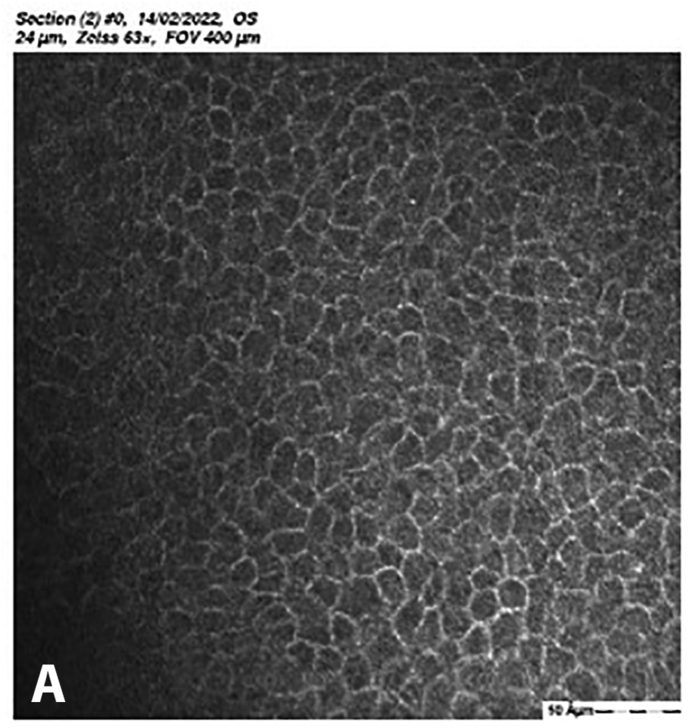

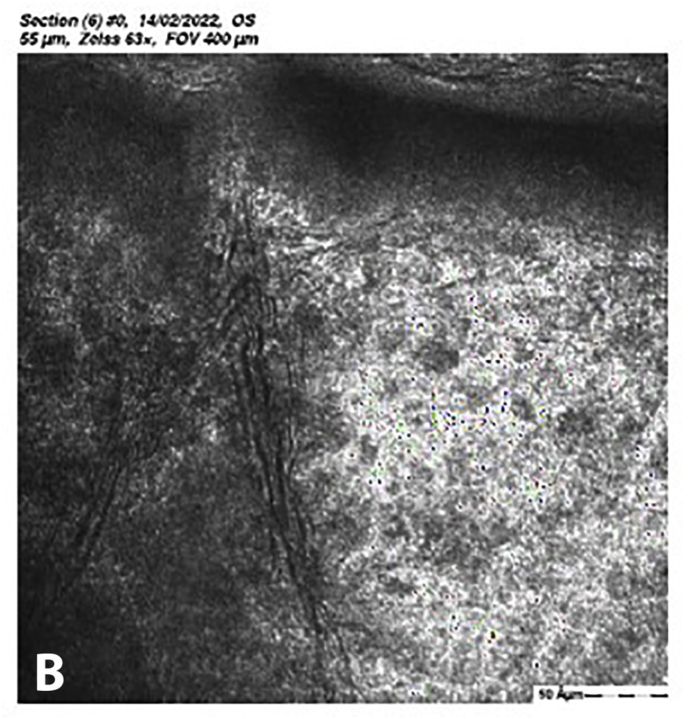

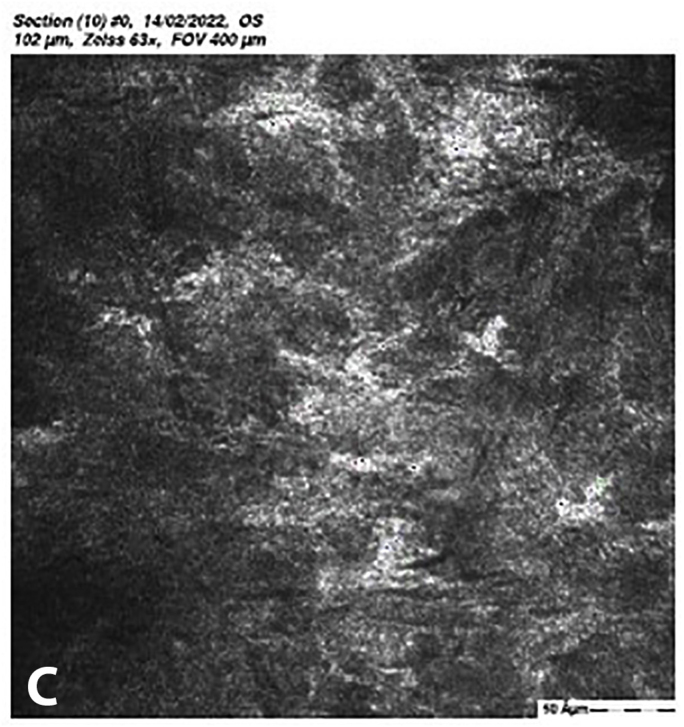

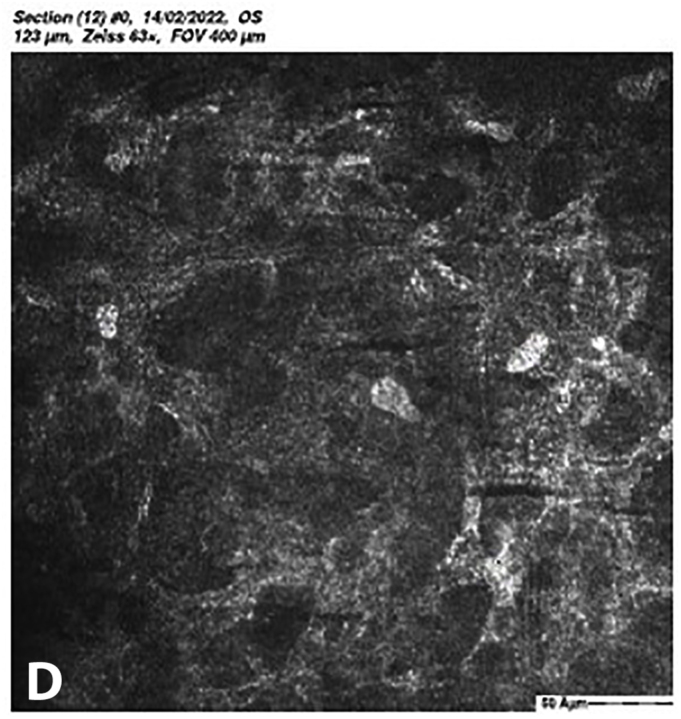

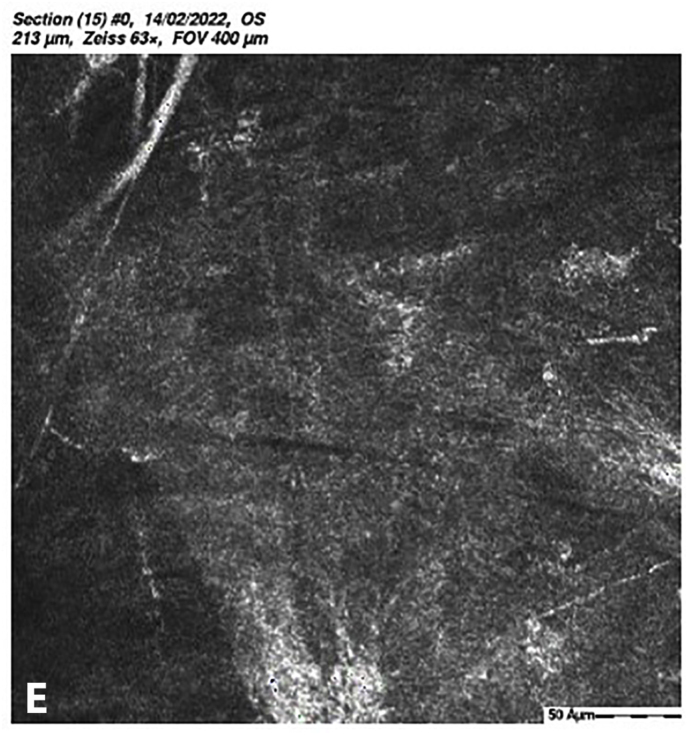

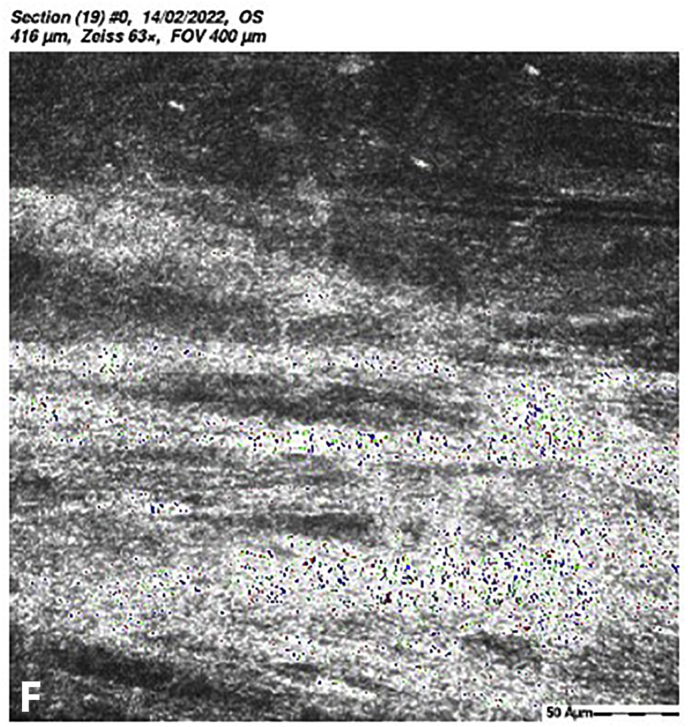

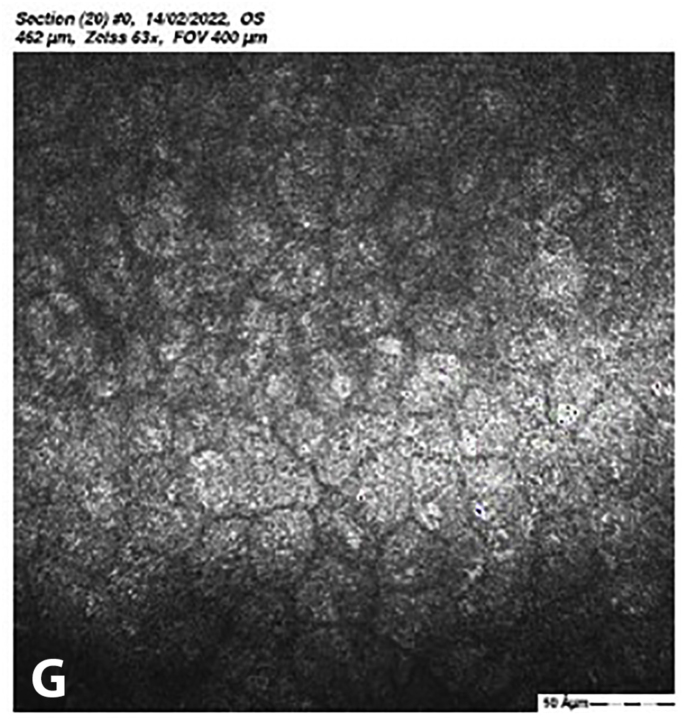

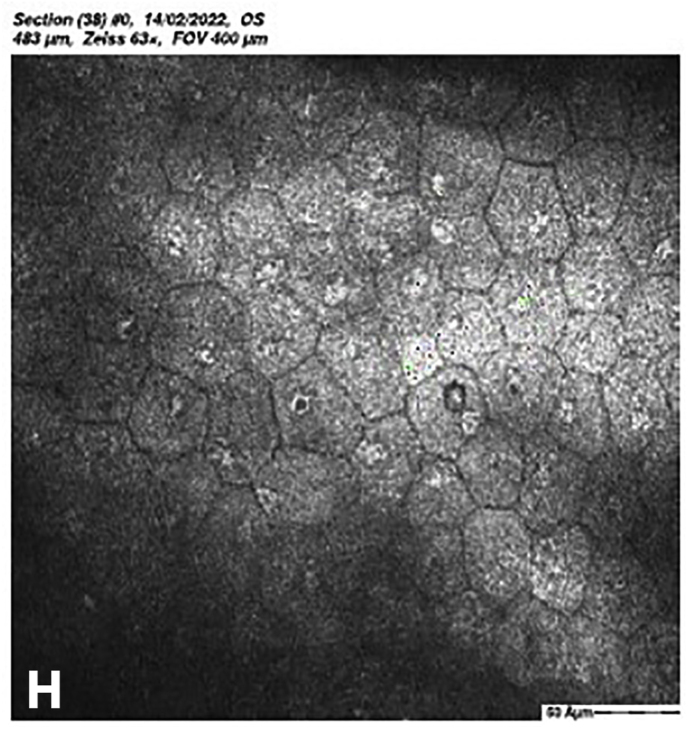
Confocal scan at 5 months post-CXL, A. normal aspect of a mosaic epithelium, B. stroma with striae, C. activated keratocytes in the stroma causing haze, D. hyper-reflective cytoplasm and extracellular lacunae, a honeycomb-like appearance, E. elongated, hyper-reflective, needle-like structures, F. hyper-reflective stromal bands, G. endothelium with reflection from stromal haze, H. visible nuclei of endothelial cells.

At 5 months post-CXL the CDVA of the left eye had improved to 20/25. The edema had spontaneously resolved, and there were some stromal folds, and pigment on the endothelium (Fig. 6b). Corneal thickness measurement had returned to 427 μm at the apex and 415 μm at the thinnest point (Supplementary Fig. 2). Confocal microscopy showed no signs of corneal stromal edema (Fig. 7).^20^

At the 6-month follow-up, slit-lamp examination showed mild haze with no effect on visual acuity. The specular microscopy measured an ECD of 1514 cells/mm^2^ in the left eye, this equals a 37 % endothelial cell loss.

## 3 Discussion

We present a case of corneal edema after customized CXL with a decrease in visual acuity at 4 months post-CXL followed by a spontaneous resolution of edema 5 months after treatment.

In general, CXL is a safe procedure with few adverse events. The most common reported adverse events are delayed healing, corneal haze, and microbial keratitis.^12^^,^^21^ Adverse events can be caused by the cytotoxic effect of the UV-A light. Studies have shown that the threshold level for damage of corneal endothelial cells is 0.65 J/cm^2^.^22^^,^^23^ The standard protocol, using a total energy of 5.4 J/cm^2^, results in radiance exposure of 0.32 J/cm^2^ at 400 μm depth. In our customized CXL protocol the radiance exposure would be 0.6 J/cm^2^ at the endothelial layer, which is still below the theoretical threshold mentioned above.

After conducting a literature review on November 23, 2023 utilizing PubMed, and Google Scholar using the key words keratoconus, crosslinking, customized/topography-guided, and adverse event/edema, we did not find any prior reports of corneal edema after customized CXL. Only a few case reports after regular CXL have been published over the years.^13^, ^14^, ^15^^,^^24^^,^^25^ According to Serrao et al. corneal edema usually happens within a month after treatment and is generally transient in nature.^12^ Possible causes of corneal edema after CXL include excessive energy delivery (due to incorrect UV-A light focus or calibration), error in corneal thickness reading during CXL, and intraoperative corneal thinning due to dehydration.^26^

The patient underwent a customized treatment protocol in the left eye with a total UV-A intensity of 10 J/cm^2^. Calibration of the UV-A light was done by the Avedro Mosaic device, which also tracks the pupil during treatment to ensure the correct area is being treated. We followed the protocol prescribed by the manufacturer, which states that corneal thickness including the epithelium should be > 375 μm. Gore et al. used the same protocol with a total energy of up to 7.2 J/cm^2^ in 700 patients (914 eyes).^27^ No cases of corneal edema were reported. The Cornea society recommends a minimum stromal thickness of 400 μm.^28^ The recommendation is based on previous research by Wollensak et al. and Spoerl et al.^22^^,^^29^ Their research showed that a minimum stromal thickness of 400 μm is necessary to prevent any endothelial damage. In our case, there was a 37 % endothelial cell loss, which suggests that a minimum stromal thickness of 375 μm is not enough to ensure endothelial safety.

After the diagnosis of corneal edema, the IT department of our hospital and the manufacturer examined the data log of the treatment, but did not reveal any errors or problems related to the calibration or tracking of the device. During irradiation, a balanced salt solution was administered to prevent the Avedro Mosaic device from losing tracking of the pupil due to corneal dehydration. To prevent dehydration, the eyelid speculum was removed during the administration of the riboflavin drops. The concentration of riboflavin could also be a safety issue.^30^ However, Beckman et al. stated the following “an increase in the concentration of riboflavin does not necessarily increase the rate of creation of free radicals; rather, a state of saturation is reached”.^5^ Recently, Abdshahzadeh et al. showed that the repeated application of riboflavin has no added value, but rather reduces the effectivity of the treatment.^31^ In 2021, Hafezi et al. published the Sub400 protocol, individualizing the fluence based on corneal thickness after riboflavin administration and before UV-irradiation.^32^ This protocol should ensure endothelial cell safety in thin corneas by lowering UV-A energy.

The patient had no complaints about a decreased vision 1 month after CXL nor did the clinical examination show any signs of corneal edema, except a slight increase in thinnest corneal thickness of 65 μm. At 4 months post-CXL, slit-lamp examination revealed corneal edema and, a wait-and-see approach was chosen. One month later the edema had resolved spontaneously. However, the question arises whether subclinical edema had escaped attention at the 1-month post-CXL visit or simply was not present. Recently, Sun et al. published a revised classification for subclinical edema based on Scheimpflug images.^33^ The criteria were validated in Fuchs Endothelial Corneal Dystrophy. Applying these classification criteria on the images 1 month post-CXL (Fig. 3) shows the presence of subclinical edema, based on the loss of so-called parallel isopachs and a displacement of the thinnest point of the cornea > 1mm from the pupil center. There was no focal posterior corneal surface depression.

The demarcation line, which is the transition zone between the crosslinked tissue and the non-crosslinked tissue,^18^ was not visible on the anterior segment OCT at 1 month post-CXL. The confocal microscopy performed 5 months post-CXL shows normal structural changes in the anterior stroma after CXL as is described in earlier studies.^20^^,^^34^ According to Jordan et al. the demarcation line is visible on confocal microscopy as a region where normal keratocytes transition into elongated, hyper-reflective, needle-like structures and then into an area of large hyper-reflective stromal bands.^20^ In the confocal microscopy images this can be seen at a depth of 416 μm, which is just above the endothelium. Normally there are no changes at the level of the endothelium since the endothelium stays unaffected during CXL.^20^^,^^34^ However, the visibility of nuclei in the endothelial cells could be an indicator of endothelial cell damage.

Based upon the research conducted by Wollensak et al. and Spoerl et al., our hypothesis is that the stromal thickness, after riboflavin soaking, was too thin.^22^^,^^29^ Moreover, considering the established UV-A threshold level for corneal endothelial cell damage, the application of 0.6 J/cm^2^ was on the higher end of the suggested energy exposure range.^22^^,^^23^ The convergence of these two factors may have contributed to endothelial cell damage, ultimately resulting in corneal edema."

## 4 Conclusion

We adhered to the safety measures prescribed by the CXL protocol provided by the manufacturer. Nonetheless, an adverse event did occur with endothelial cell loss. To avoid adverse events and to be able to safely execute a CXL treatment it is important to have an extensive preoperative clinical examination and imaging of the corneal topography as well as endothelial cell density, and to have a clear CXL protocol respecting the safety boundaries. We recommend either adhering to a minimal stromal thickness of 400 μm before administering UV-A irradiation, using a contact lens or adjusting the irradiation to prevent this complication.

## Patient consent Statement

The patient gave written informed consent for this publication.

## Funding

No funding

## CRediT authorship contribution statement

**Magali M.S. Vandevenne:** Methodology, Investigation, Conceptualization, Data curation, Formal analysis, Visualization, Writing – original draft, Writing – review & editing. **Tos T.J.M. Berendschot:** Writing – review & editing. **Nienke Visser:** Writing – review & editing. **Mor M. Dickman:** Writing – review & editing. **Rudy M.M.A. Nuijts:** Conceptualization, Supervision, Writing – review & editing.

## Declaration of competing interest

M.M. Dickman: Carl-Zeiss: consultant.

R.M.M.A. Nuijts: Alcon: consultant, research grant; 10.13039/100005565Johnson & Johnson: consultant; Théa Pharma: consultant; 10.13039/100007569Carl-Zeiss: consultant; Teleon: research grant

The other authors have no financial disclosures or competing interests.

## Figures and Tables

**figs1:**
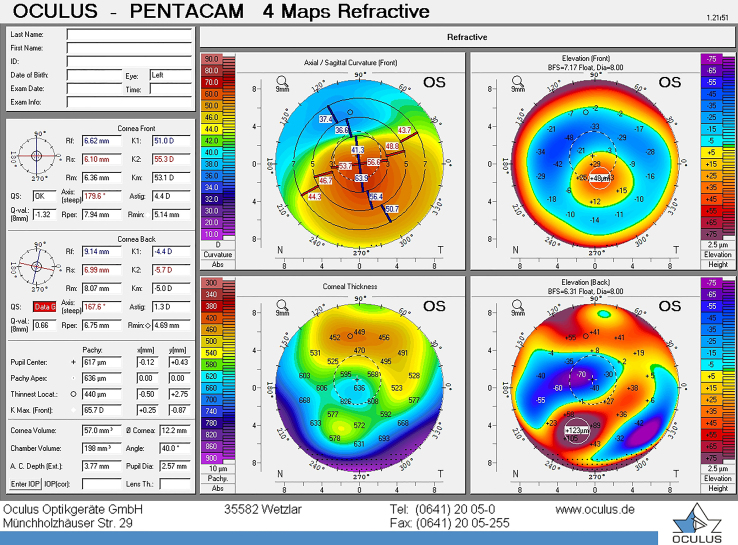


**figs2:**